# Dietary Habits of Saharawi Type II Diabetic Women Living in Algerian Refugee Camps: Relationship with Nutritional Status and Glycemic Profile

**DOI:** 10.3390/nu12020568

**Published:** 2020-02-22

**Authors:** Alessandro Leone, Alberto Battezzati, Sara Di Lello, Stefano Ravasenghi, Babahmed Mohamed-Iahdih, Saleh Mohamed Lamin Saleh, Simona Bertoli

**Affiliations:** 1 International Center for the Assessment of Nutritional Status (ICANS), Department of Food, Environmental and Nutritional Sciences (DeFENS), University of Milan, Via Sandro Botticelli 21, 20133 Milan, Italy; alberto.battezzati@unimi.it (A.B.); stefano.ravasenghi@unimi.it (S.R.); simona.bertoli@unimi.it (S.B.); 2 Movimento Africa 70 NGO, Via Missori 14, 20900 Monza, Italy; sara.dilello71@gmail.com; 3 Ministerio de Desarrollo Económico, República Árabe Saharaui Democrática, 37000 Tindouf, Algeria; bamief@yahoo.es; 4 Ministerio de Salud Pública, República Árabe Saharaui Democrática, 37000 Tindouf, Algeria; salehmohamedlaminsaleh@gmail.com; 5 Istituto Auxologico Italiano, IRCCS, Lab of Nutrition and Obesity Research, 20145 Milan, Italy

**Keywords:** dietary habits, diabetes, Saharawi refugee camps, obesity, nutritional status, dietary pattern, food consumption, glucose, insulin, HbA1c

## Abstract

Diabetes is one of the main health problems among Saharawi refugees living in Algerian camps, especially for women. As is known, diet plays an important role in the management of diabetes. However, the dietary habits of Saharawi diabetic women are unknown. Therefore, we investigated the dietary habits and established their relationship with the nutritional status and glycemic profile of such women. We recruited 65 Saharawi type II diabetic women taking orally glucose-lowering drugs only. Dietary habits were investigated using qualitative 24 h recall carried out over three non-consecutive days. Anthropometric measurements were taken and blood parameters were measured. About 80% of the women were overweight and about three out of four women had uncompensated diabetes and were insulin resistant. The Saharawi diet was found to mainly include cereals, oils, sugars, vegetables (especially onions, tomatoes, and carrots), tea, and meat. Principal component analysis identified two major dietary patterns, the first one “healthy” and the second one “unhealthy”. Women in the higher tertile of adherence to the unhealthy dietary pattern had a higher homeostatic model assessment for insulin resistance (HOMA) index (b = 2.49; 95% CI: 0.41–4.57; *p* = 0.02) and circulating insulin (b = 4.52; 95% CI: 0.44–8.60; *p* = 0.03) than the women in the lowest tertile. Food policies should be oriented to improve the quality of diet of Saharawi diabetic women.

## 1. Introduction

Type II diabetes is a chronic condition characterized by high levels of glucose in the blood and is caused by a high resistance to the effects of insulin or to insufficient insulin production. The likelihood of developing type II diabetes depends on a combination of genetic and environmental factors such as lifestyle and diet. As of 2015, worldwide prevalence of type II diabetes was estimated as being present in 392 million people (6% of the world’s population) [1]. 

Many factors, including the changing pattern of food consumption, urbanization, physical inactivity, and perinatal under-nutrition, have led to a rapid increase in the prevalence of type II diabetes mellitus in developing countries over the last decade [2]. However, the quality of diabetes care in many developing countries has not increased as quickly, and currently still remains poor [3,4]. This is because of the unavailability, or high cost, of diagnostic tests and diabetes monitoring, including HbA1c, glucose-lowering drugs, and insulin, as well as poorly trained medical doctors and paramedics, and poor compliance with the therapy and follow-up. Moreover, if it is true that diet plays an important role in the management of diabetes [5,6], it is also true that, in some developing countries, due to war, political issues, poverty, and adverse environmental conditions, there may be barriers to accessing food, particularly food of high nutritional quality. The situation is exacerbated among refugees, who, in addition to these problems, also face the stress of changing living and dietary habits [2]. 

Since 1975, the Saharawi population has been living in refugee camps located in the Algerian desert in the southwest of the country. Their survival is guaranteed by the food rations and food supplements provided by the international community [7,8]. Monthly, the World Food Programme provides 90,000 family rations including cereals, pulses, vegetable oil, sugar, and super-cereal. In the camps, there are also local dealers who sell food products including rice, pasta, sugar, oil, juice, tea, milk, water, eggs, biscuits, and canned food. Seasonal fruits and vegetables are also available, but their consumption is low [9] and it is not clear which varieties are actually consumed and how often. Thus, it is not surprising that, in this food context characterized by high density and starchy foods, obesity and diabetes are the main health problems among refugees [10,11]. Although the prevalence of diabetes is still unclear, recent surveys report percentages ranged from 7% to 27% for diabetes and ranged from 10% to 15% for prediabetes [11,12]. The risk seems to be greater in women than in men [11]. These data are worrying, especially when compared to the prevalence of diabetes in the population of Western countries, where the prevalence ranges from 5% to 10% [13,14]. It is therefore important to know the food consumption of diabetic subjects in order to identify and correct the unhealthy dietary habits and to plan food education campaigns for patients, local doctors, and healthcare workers. 

Our study aimed at investigating the dietary habits of Saharawi type II diabetic women living in the Algerian refugee camps, and establish a relationship with the nutritional status and the glycemic profile of the female refugee population.

## 2. Materials and Methods

### 2.1. Study Design and Subjects

We carried out a cross-sectional study on 72 Saharawi type II diabetic women living in the dairas (towns) of Edchedería, Bir Lehlu, Mahbes, and Farsia at the wilaya (province) of Smara, and in the dairas of 27Febrero and Lemsid at the wilaya of Bojador. To be included in the study, the subjects had to have been diagnosed as type II diabetes for at least 1 year, and had to be free of advanced retinopathy, terminal renal failure, active diabetic ulcers, amputations, and heart failure; not undergoing insulin therapy; and being treated only with oral hypoglycemic agents. Women were randomly selected from among the population of diabetic women, followed by the dispensary of each daira. Recruitment took place in November 2019. On the first investigation day, all subjects underwent blood testing and the measurement biochemical parameters, and the results were made available during the clinical investigation. On the same day, we conducted a focus group with six Saharawi women in order to explore Saharawi gastronomic traditions. Over the following days, each subject was subjected to a clinical investigation, an anthropometric assessment, and dietary habit assessment. The present study was conducted according to the guidelines laid down in the Declaration of Helsinki. The Ministerio de Salud Pública (Ministry of Public Health) of Saharawi Arab Democratic Republic approved the study procedures (agreement 10 November 2018), and each subject gave written informed consent to participate in the study.

### 2.2. Clinical Investigation

An Italian-speaking physician accompanied by a Saharawi Arabic- and Italian-speaking physician as an interpreter conducted the clinical investigation through a structured interview. Clinical history of diabetes (years passed from the diagnosis and past and current drug therapies), the presence of micro- and macrovascular comorbidities, and the co-presence of other diseases such as dyslipidemia, hypertension, hypo- and hyperthyroidism, celiac disease, and anemia were investigated. For ethical reasons, if the current drug therapy was deemed inadequate to treat the current diabetic status, it was changed, but the one used until the day of the investigation was included in the dataset. Finally, blood pressure was measured using a random-zero mercury sphygmomanometer following the Joint National Committee 7 guidelines [15]. If systolic or diastolic blood pressure was ≥130/85 mm Hg, blood pressure was measured in other two different occasions. Women were defined as suffering of hypertension if they were prescribed with antihypertensive drugs or if systolic or diastolic blood pressure was found as being ≥130/85 mm Hg in three consecutive occasions.

### 2.3. Anthropometric Assessment

A physician with long-term experience as an anthropometrist took the anthropometric measurements following international guidelines [16]. Body weight was measured to the nearest 100 g with a column scale and with participants wearing only light underwear, which was performed after bladder emptying. Body height was measured to the nearest 0.1 cm using a vertical stadiometer. BMI was calculated as weight (kg)/height (m)^2^ and overweight and obesity were classified following the NIH guidelines [17]. Waist (WC) and hip circumferences (HC) were measured with a non-stretch tape to the nearest 0.5 cm at the midpoint between the last rib and the iliac crest and around the widest portion of the buttocks, respectively. Waist-to-hip ratio (WHR) was calculated. High WC was defined as WC ≥ 80 cm, the threshold value for abdominal obesity proposed for sub-Saharan women [18,19]. Skinfold thicknesses were measured using a Holtain Tanner/Whitehouse Skinfold Calliper (Holtain Ltd.). Four skinfolds were measured: biceps, triceps, subscapular, and suprailiac. Each skinfold was measured three times and the mean was used for analysis. When the thickness of a skinfold was >40 mm, it was put as missing in the dataset as it was not measurable. Body density was estimated using the most appropriate Durnin and Womersley’s formula [20], depending on the measurable skinfolds. Body fat (BF, %) was estimated from body density using Siri’s [21] formulas.

### 2.4. Dietary Assessment

The day before starting the investigation, we explored, using a focus group of six Saharawi women, Saharawi gastronomic traditions and meal preparation methods, and identified known dietary items potentially consumed in Saharawi families. After this preliminary activity, we investigated the food consumption of Saharawi diabetic women over three non-consecutive days using qualitative 24 h recall. We considered two working days and a public holiday. We recorded all the food directly consumed and all the ingredients used in the individual recipes. The interviews were conducted by an Italian- and Spanish-speaking researcher accompanied by a Saharawi Arabic- and Spanish-speaking agronomist and PhD as the interpreter. The foods recorded during the three-day investigation were gathered together in the following food groups: cereals and derivatives, tubers, vegetables, fruit, legumes, oils and fats, meat, fish, eggs, milk and dairy products, nuts, sugars and sweets, soft drinks, and tea and coffee. Being a qualitative investigation, in the data analysis the number of occasions during which each food item and food group was consumed over the three days was considered. We considered a food group always consumed if eaten at least once a day for three days, often consumed if eaten at least once a day for two days out of three, sometimes consumed if eaten at least once a day for only one day out of three, and never consumed if never eaten during the three days of investigation. 

A posteriori dietary patterns were derived by the frequencies of consumption of each food item and food group using principal component analysis (PCA). Food items were classified into 20 predefined food groups, on the basis of the similarity of nutrient profiles or culinary usage. The orthogonal rotation (rotate with varimax option) was used to derive optimal non-correlated components facilitating the interpretability. The number of dietary patterns to retain was defined on the basis of eigenvalues >2.0 [22,23]. To characterize each dietary pattern, we considered factor loadings with an absolute value ≥0.300 [24,25]. For each dietary pattern, factor scores were obtained and categorized by tertiles (T1 = low adherence, T2 = medium adherence, T3 = high adherence); the lowest tertile (T1) of each dietary pattern was used as the reference for further analyses.

### 2.5. Laboratory Evaluations

On the day before starting the investigation, a blood sample was obtained from each subject and sent to the National Hospital laboratory (Rabouni, Saharawi refugee camps, Algeria) for analysis. Glucose, triglycerides, total cholesterol, alanine transaminase (ALT), and aspartate aminotransferase (AST) were measured by means of an enzymatic method (Mindray BS 230 clinical chemistry analyzer, Shenzhen, China). Insulin was measured using a sandwich ELISA test (BioVendor, Brno CZ). Finally, HbA1c was measured using a spectrofluorimetric method (Quo-Lab HbA1c, EKF Diagnostics, Cardiff, United Kingdom). The analysis was conducted by Saharawi laboratory technicians under the supervision of an Italian laboratory technician. Impaired fasting glucose was defined as glucose ≥126 mg/dL and insulin-resistance as homeostatic model assessment for insulin resistance (HOMA) index ≥2.5. As a glyceamic target for a reduced risk of micro and macro vascular complications, we used a HbA1c target of <7.0% [26].

### 2.6. Statistical Analysis

Several continuous variables had a non-Gaussian distribution, and thus they are reported herein as 25th, 50th, and 75th percentiles. Discrete variables are reported as numbers and percentages. Linear regression was used to analyze the relation between dietary patterns and the variables regarding nutritional status and glycemic profile. Age (continuous, years), years passed from the diagnosis of diabetes (continuous, years), and BMI (only when we investigated the relation between dietary patterns and glycemic profile, continuous, kg/m^2^) were included in the model as covariates. Tertiles of dietary pattern were also included in the models, and the lowest one was used as a reference. A *p*-value < 0.05 was considered statistically significant. Statistical analysis was performed using STATA version 12.0 (StataCorp, College Station, TX, USA). 

## 3. Results

Of the 72 women initially recruited, 7 withdrew their consent to participate to the study and did not show up on the day of the blood sampling. Therefore, all analysis was conducted on a sample of 65 women.

### 3.1. Socialdemographic Characteristics

Our sample included 65 diabetic women from six dairas of two different wilayas. Median age was 58 years (25th–75th: 52–64 years). The sociodemographic characteristics of the sample are reported in Table 1. 

A total of 58.5% of the women were married. Overall, the women lived in families consisting of 6 people (25th–75th: 5–8 people) to a maximum of 16 people. The education level was generally low—38.5% of the women had had no education at all, and 27.7% had received only a primary education; only 3.1% of the women had an academic title. Finally, half of the recruited women worked, whereas the other 50% were housewives or retired.

### 3.2. Clinical History, Nutritional Status, and Metabolic Profile

The women were all affected by type 2 diabetes that had been diagnosed for at least a year (median: 6 years, 25th–75th: 3–11 years). At the time of recruitment, 38.5% of them were being treated with metformin, 21.5% with glibenclamide, 33.9% with a combination of metformin and glibenclamide, and 6.2% with other therapies including sitagliptin and glimepiride alone or in combination with metformin. Moreover, 69.2% of women were affected by hypertension, 24.6% by dyslipidemia, and only one woman suffered of hypothyroidism. No women were affected by celiac disease and hyperthyroidism. Finally, 46.2% of women had suffered from anemia during previous pregnancies. However, at the time of recruitment, only 15.4% of women had hemoglobin values < 12 g/dL.

The nutritional and metabolic characteristics of the sample are reported in Table 2.

With a median weight of 71.7 kg and a median height of 157 cm, median BMI was 28.7 kg/m^2^ (25th–75th: 25.6–31.8 kg/m^2^). Using the cut-off proposed by the NIH for general obesity, 20.0% of women were normal weight, 35.4% were overweight, and 44.6% were obese, including four women (6.2%) with a second class of obesity and one woman (1.5%) with a third class of obesity. Total body fat, with a median value of 45.4% (25th–75th: 43.7–47.3%) was high, and all women exceeded 30% of body fat. Concerning the abdominal obesity, all women had an elevated waist circumference, and 58.5% had a WHR ≥1.

As for the glycemic profile, only 10.8% of women reached the glycemic target. Insulin-resistance was found in the 75.4% of women. Finally, 93.9% of women had a fasting glucose ≥ 126 mg/dL.

### 3.3. Dietary Habits

**Figure 1:**
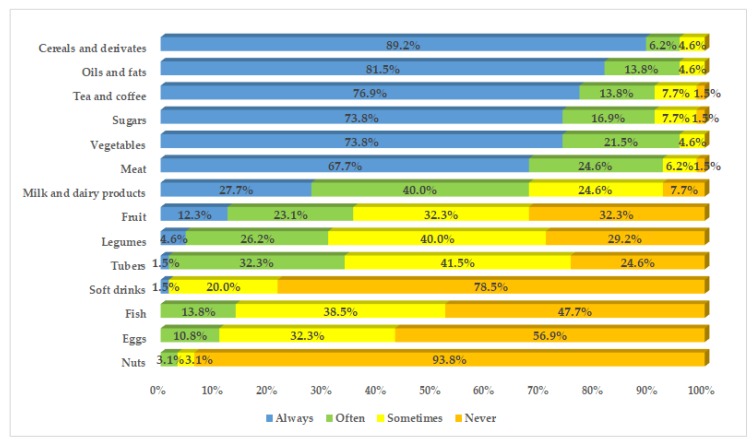
Propensity of the Saharawi diabetic women to the consumption of the different food groups.

Figure 1 reports the propensity of the recruited women to the consumption of the different food groups. 

In the diet of Saharawi diabetic women, it is common to consume cereals and derivatives, oils and fats, tea and coffee, sugars, vegetables, and meat. On the other hand, the consumption of milk and dairy products, fruits, legumes, and tubers is moderate. Fish, eggs, and soft drinks are not heavily consumed, whereas the consumption of nuts is almost completely absent.

**Figure 2:**
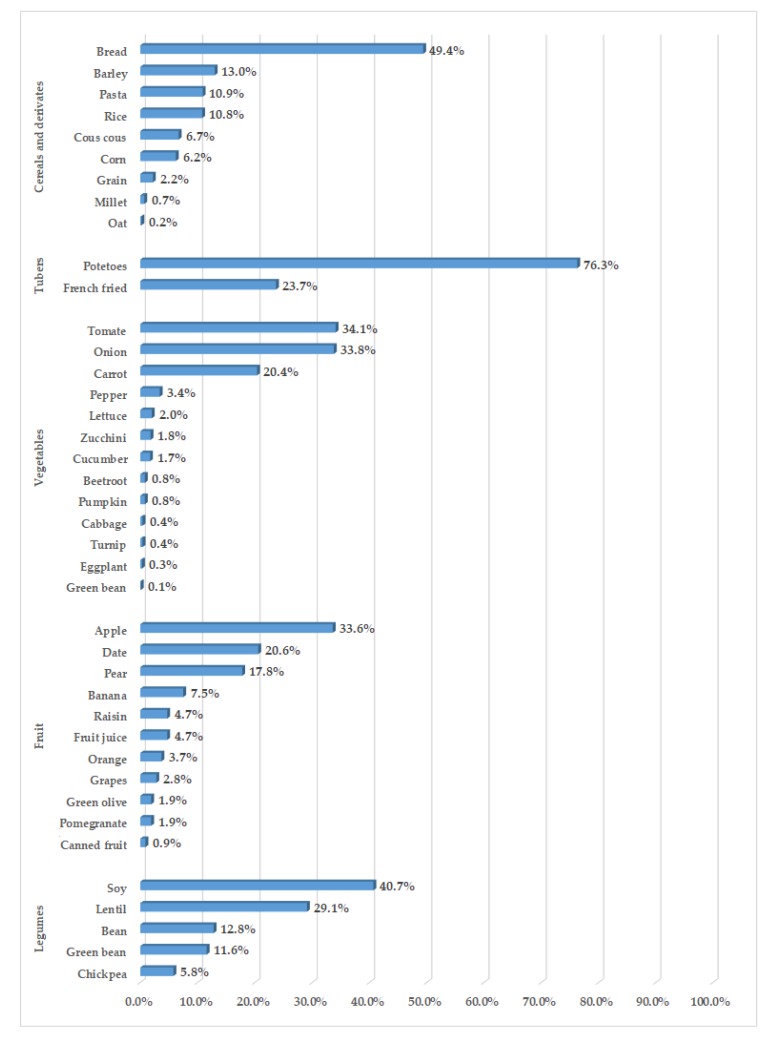

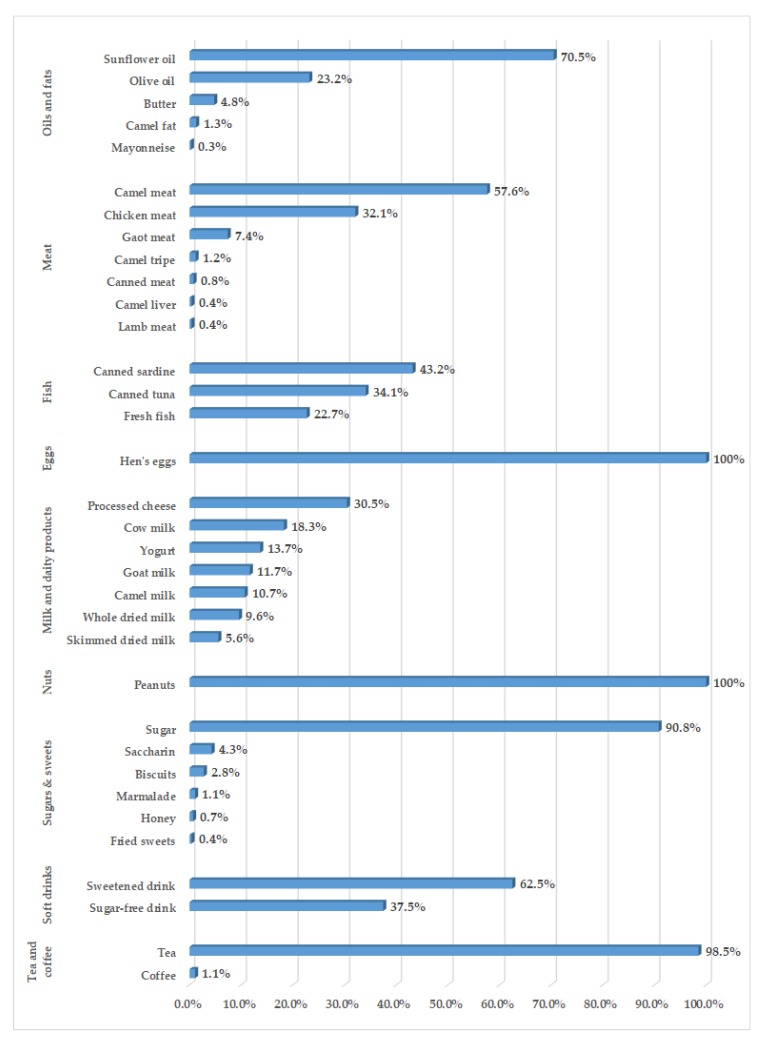
Frequency of consumption of each food item within each food group category.

Figure 2 reports the frequency of consumption of each food item within each food group category. During the 3 days of the investigation, we identified 74 food items. However, only 34 had a consumption frequency such as to represent at least 10% of their own food group. Wheat bread was the most consumed food in the cereal and derivatives category, followed by the consumption of some unprocessed cereals and other cereal derivatives. Among the unprocessed cereals, Saharawi diabetic women consumed barley, used to produce a soup called “ensha”, prepared by adding water and sunflower oil or olive oil and consumed mainly at breakfast; rice, used to accompany meat dishes; and corn, used in the form of flour for the production of a typical local drink, called “gofio”, obtained by dissolving corn flour in water or milk, sometimes with the addition of sugar. The consumption of other unprocessed cereals was minor. 

Among the cereal derivatives, the Saharawi diabetic women consumed bread, followed by pasta and cous cous, to accompany meat and fish dishes. About 88% of the vegetable consumption was limited to onions, tomatoes, and carrots, used almost exclusively in cooked form, to prepare stewed meat dishes or legume soups. The remaining 12% was made up of other vegetables, also used mainly in cooked form, consumed very occasionally. The consumption of green leafy vegetables was practically absent. As for fruit consumption, in 33.6% of cases it was apples, in 20.6% of cases it was fresh or dried dates, in 17.8% of cases it was pears, and in 7.5% of cases it was bananas. Other fruits were consumed only occasionally. Soy was the most consumed legume among diabetic Saharawi women (40.7% of cases), followed by lentils and beans, used to produce legume soups, and more occasionally by peas and chickpeas, used as an ingredient in some meat dishes. Sunflower oil was used in about 70% of occasions, and olive oil only one time out of four. Moreover, the consumption of animal fats was minor. In 57.6% of the cases in which meat was consumed, it was represented by camel meat, followed by chicken meat in 32.1% of the cases and by goat meat in 7.4%. Fish was represented by canned sardines and canned tuna in the 77.3% of occasions in which fish was consumed. Only in about one occasion out of four was fresh fish consumed. As for the consumption of milk and dairy products, in 30.5% of the occasions it was processed cheese (triangle cheese), the only cheese that can be found in Saharawi refugee camps, followed by cow’s milk, yogurt, goat and camel milks, and finally by powdered milk. Eggs and nuts were represented exclusively by hen’s eggs and peanuts, respectively. Finally, the sugar and sweet category was almost entirely represented by the sugar used to sweeten tea, a beverage highly consumed by Saharawi diabetic women. 

### 3.4. Relationship between Dietary Habits and Nutritional and Metabolic Status

We identified two major dietary patterns with eigenvalues ≥2.0, explaining approximately 30.0% of the total variance among the 20 food groups (Table 3). The first, characterized by a more frequent consumption of vegetables other than onions, tomatoes, and carrots; olive oil; fresh fruit; white meat; eggs; milk and dairy products; and legumes and by a less frequent consumption of animal fats and red meat was defined as a healthy dietary pattern. The second one, characterized by a more frequent consumption of refined cereals, vegetables (especially cooked), sunflower oil, dried fruit, red meat, sugar, sweets, and carbonated beverages and by a less frequent consumption of fresh fish was defined as an unhealthy dietary pattern. 

When we explored the association of dietary patterns with the nutritional status and glycemic profile (Table 4), we found that subjects in the higher tertile of adherence to the unhealthy dietary pattern tended to have more body fat (b = 1.9; 95% CI: −0.12, 3.91; *p* = 0.064) than participants in the lower tertile. Moreover, they had higher values of HOMA index (b = 2.49; 95% CI: 0.41, 4.57; *p* = 0.02) and serum insulin (b = 4.52; 95% CI: 0.44, 8.60; *p* = 0.03) compared to the subjects in lowest tertile of adherence.

## 4. Discussion

In this study, we investigated the dietary habits of Saharawi type II diabetic women living in the Algerian refugee camps, and related them to their nutritional status and glycemic profile.

We observed that the nutritional and metabolic situations of type II diabetic women living in the refugee camps was quite critical. The prevalence of overweight and obesity (80.0%) was higher than that found in the Saharawi women of the general population [10]. Moreover, all the women were characterized by central obesity. The low nutritional quality, as well as the Saharawi tradition of associating large body sizes with well-being and beauty, have been suggested as major factors associated with the increased prevalence of overweight and obesity [10,27,28]. Another factor is the high level of physical inactivity among Saharawi women [29]. In our sample, about 50% of the women did not carry out any activity within the Saharawi community, and this led them to spend more time seated, thereby reducing their energy expenditure. Last but not least, it must be said that many of the foods consumed by the Saharawi population arrive with humanitarian aid, and are packed in cans. Moreover, the refugee camps have problems associated with waste disposal—it is not uncommon to come across open-air landfills a short distance from camps, where different types of food packaging have been abandoned. This could expose the population to high levels of bisphenol A (BPA), an endocrine disruptor associated with the risk of obesity and diabetes [30,31,32] and mainly found in food packaging such as polycarbonate containers and the epoxy resins that cover the inner wall of cans [33]. Indeed, BPA has been found to interfere with the production, release, transport, metabolism, binding, action, or elimination of natural hormones, producing the so called “diabesity phenotype” [34]. It is then possible that high BPA exposure levels may be hidden behind the high prevalence of obesity and diabetes found in the Saharawi population. 

As for the glycemic profile, 9 out of 10 women did not reach the glycemic target for a reduced risk of micro and macro vascular complications. Moreover, 75.4% of subjects were insulin-resistant, a situation presumably the consequence of obesity itself, but also of inadequate diabetes care and a dietary pattern characterized by little variability and poor nutritional quality. As in other developing countries [2], the Saharawi refugee camps also have a poor supply of glucose-lowering drugs and insulin, as well as materials, reagents, and tools for diabetes diagnosis and monitoring. In our sample, almost all of the diabetic women were treated with metformin, glibenclamide, or a combination of the two. However, during the clinical investigation, we observed, in some cases, poor compliance with the drug therapy, as well as a lack of monitoring and follow-up. With regard to the diet of Saharawi diabetic women, as reported for the general population [9], it was characterized by low variability and diversity. Overall, the diet consisted mainly of cereals and derivatives, oils and fats, tea, sugars, vegetables, and meat. Among the cereals and derivatives, wheat bread prevailed, followed mainly by barley, rice, and couscous. The consumption of vegetables was frequent, but the diversity of the vegetables consumed was very low, with tomatoes, onions, and carrots representing about 90% of the variability of consumed vegetables. Other vegetables were only minimally consumed. It is likely that in a context of low economic availability, resources will be allocated to food considered as being more nutritious and rewarding. This would justify the high consumption of meat and the preference for sunflower oil rather than olive oil. A recent study conducted on the general population of Saharawi observed that 67% of the population live without an income, whereas 9% had a monthly income of up to 30 euros and 22% above 30 euros. From this, we can understand the difficulty of finding medicine and food of high nutritional quality when they are not provided by the local government, non-governmental organizations, or the international community [9]. Frequent and widespread in the Saharawi diet was the consumption of sweetened tea. The consumption of milk and dairy products was moderate, as well as the consumption of fruit, legumes, and tubers. Only occasionally included in the Saharawi diet were eggs, fish, and soft drinks. A parenthesis regarding fish—three quarters of consumption was canned fish. Finally, nuts were almost completely absent.

When we investigated the relation between dietary habits and nutritional status and glycemic profile, we first explored which dietary patterns best described the dietary habits of Saharawi diabetic women. This was because the approach based on studying the dietary pattern, differently from the use of a single nutrient of food, allows account to be taken for the synergistic and/or antagonistic interactions among nutrients [35,36,37]. Moreover, findings obtained using overall dietary patterns are more amenable to translation into public health practice and food politics [35]. We identified two major dietary patterns: one, which we defined “healthy”, characterized by a more frequent consumption of the vegetables minimally consumed, olive oil, fresh fruit, white meat, eggs, milk and dairy products, and legumes, and by a less frequent consumption of animal fats and red meat; the other, which we defined “unhealthy”, characterized by a more frequent consumption of refined cereals; vegetables such as onions, tomatoes, and carrots; sunflower oil; dried fruit; red meat; sugar; sweets; and carbonated beverages, and by a less frequent consumption of fresh fish. We did not find any association between the adherence to the healthy dietary pattern and the nutritional status and the glycemic profile of Saharawi diabetic women. Several randomized controlled trials and prospective studies have shown an improvement in the glycemic profile, including a reduction in HbA1c levels in diabetic patients after implementation of a medical nutritional therapy [38,39,40]. It must be said that the situation in which Saharawi diabetics find themselves is more critical, with higher HbA1c values, prescribed drug therapy often being insufficient and subjected to continuous different variations by medical staff each time, low drug treatment compliance, and glycemic checks and follow-up rarely performed. Therefore, in our opinion, this is not a failure of the diet, but rather an inability of the diet, on its own, to improve such a critical context. In contrast, increased adherence to the unhealthy dietary pattern was associated with higher values of HOMA index and circulating insulin. The most accredited hypothesis is that the high intake of refined carbohydrates, particularly sugars from sweetened tea, are the main culprits of these associations. According to local tradition, the tea consumption among refugees is three servings, each 30 mL, three times a day [41]. We asked the women in the focus group to prepare tea according to their traditions and habits. By weighing the amount of sugar put in the teapot and counting the number of small glasses of tea obtained, we found that each 30 mL glass of tea contained about 12 g of sugar. Thus, it is not surprising that a dietary pattern characterized by high sugar intake, and therefore continuous stimulation of pancreatic insulin secretion, can, over time, lead to increased insulin resistance and hyperinsulinemia. What is really surprising is that the consumption of such highly sweetened tea is very frequent among the Saharawi population, even though these women are diabetic. The most likely hypothesis is that there is little awareness among Saharawi women of the negative effects of such high sugar consumption on the glycemic profile of an individual, especially if diabetic. Therefore, nutritional education campaigns should be undertaken to give women instructions and recommendations for a good dietary management of diabetes. At the same time, the donated food rations, initially designed to deal with an acute emergency situation, are unsatisfactory for 45 years of life in exile in the desert. Such rations cannot have as their exclusive nutritional objective the satisfaction of daily caloric intake; they must also meet the needs of macro- and micronutrients. On the basis of this, the food rations should be modified to include fresh food such as fruit and vegetables and to replace refined starchy foods and sugar with whole foods and low glycemic index foods.

Therefore, we believe that there are several points on which to act in order to improve the living conditions of these people: (1) to improve diabetes management through the training of local health personnel and providing resources (funding, materials, and tools) to allow diabetic diagnostic tests and monitoring; (2) to promote an active lifestyle and good dietary habits through education programs aimed at the diabetic population, evaluating their effectiveness through prospective studies; (3) to modify the food rations donated by the international community, replacing sugar and refined foods with fresh and low glycemic index foods; and (4) to finance projects aimed at increasing the local food production, thus ensuring not only fresh locally produced foods that increase the dietary variability, but also work for the population, as well as evaluating their impact on dietary habits through monitoring surveys. This is the first study that describes the dietary habits of Saharawi diabetic patients and relates them to their nutritional status and glycemic profile. The strengths of this study are numerous. Food consumption was investigated over three different days, including public holidays, thus giving a good representation of Saharawi dietary habits and the glycemic profile was fully measured. The subjects were recruited from different dairas to achieve an overall representation of the Saharawi population.

However, the study is not free of limitations. The analysis of food consumption was only qualitative and not quantitative. Consequently, the data reported refer to consumption frequency, without taking into account the quantities actually consumed. This method was chosen because the quantification of the food consumed was practically impossible for Saharawi women. Traditionally, in Saharawi families, all the family members eat together, taking food from a single plate. Thus, in large families, where there can be as many as 16 members between adults and children, it is difficult to quantify how much of each food each individual ate. The estimates would have been very imprecise, and it was therefore decided to use only food frequency, a choice that did not prevent dietary patterns from being defined. Moreover, we only recruited women. This was mainly for three reasons: (1) obesity and diabetes are more prevalent in women; (2) as our data show, women in the Saharawi community often do not work and have more free time than men, thus facilitating the recruitment; and (3) in the Saharawi family, meals are prepared by women, thereby they have a greater knowledge about foods and ingredients used in different food preparations. This, however, limits the generalizability of our results on the male diabetic population, on which confirmatory studies must be carried out. Finally, we excluded cases with micro- and macrovascular complications, as it is likely that they have had poor control of diabetes combined with poor dietary habits for a long period (years). In these subjects, the effect of the diet on the glycemic profile might be weaker in the absence of a correct and perpetrated pharmacological therapy. In addition, it is possible that these patients may have received additional dietary recommendations (e.g., protein intake in subjects with diabetic nephropathy) that may affect dietary habits and have a different effect on the glycemic outcome. Therefore, we preferred to recruit women so that they were as homogeneous as possible, in order to limit the statistical noise.

## 5. Conclusions

The diet of Saharawi type II diabetic women is characterized by little variability. Indeed, a dietary pattern rich in starches and sugar-rich foods, such as the diet of the Saharawi diabetic women, appears to be associated with increased insulin resistance and hyperinsulinemia. Thus, food policies aimed at improving dietary quality should be undertaken by the local government, whereas, at the same time, the international community’s provision of food should include foods that are better suited to dealing with the long term situation that has now been chronic for 45 years.

## Figures and Tables

**Table 1:** Sociodemographic characteristics of the recruited women.

		*n*	%
Wilaya (Province)	Daira (Town)		
Smara		44	67.7
	Eydaria	12	18.5
	Bir Lehlu	10	15.4
	Mahbes	11	16.9
	Farsia	11	16.9
Bojador		21	32.3
	27Febrero	10	15.4
	Lamsid	11	16.9
Education			
None		25	38.5
Primary level		18	27.7
Secondary level		20	30.8
University		2	3.1
Marital status			
Unmarried		1	1.5
Married		38	58.5
Divorced		12	18.5
Widow		14	21.5
Occupation			
Housewife		29	44.6
Retired		4	6.2
Worker		32	49.2

Abbreviation: *n* = number of subjects for each category.

**Table 2:** Nutritional and metabolic characteristics of the recruited women.

	P25	P50	P75
Nutritional status			
BMI (kg/m^2^)	25.6	28.7	31.8
Waist circumference (cm)	97.0	101.8	108.5
Hip circumference (cm)	95.8	101.0	108.5
Waist-to-hip ratio	0.9	1.0	1.1
Arm circumference (cm)	31.0	34.0	36.0
Tricipital SKF (mm)	30.0	34.0	41.0
Bicipital SKF (mm)	21.0	25.5	33.0
Subscapular SKF (mm)	32.5	36.0	41.0
Suprailiac SKF (mm)	32.0	36.5	41.0
Body fat (%)	43.7	45.4	47.3
Metabolic parameters			
Glucose (mg/dL)	163	207	243
Insulin (mU/mL)	4.9	8.8	13.9
HOMA	2.4	4.5	7.1
HbA1c (%)	7.9	9.4	10.3
Systolic blood pressure (mm Hg)	120	135	145
Diastolic blood pressure (mm Hg)	75	80	89
Total cholesterol (mg/dL)	165	188	219
Triglycerides (mg/dL)	98	126	166
AST (U/L)	20	24	30
ALT (U/L)	17	21	26
Hemoglobin (g/dL)	12.7	14.0	14.6

Abbreviations: P25 = 25th percentile; P50 = 50th percentile; P75 = 75th percentile.

**Table 3:** Score coefficients derived from principal component analysis regarding foods or food groups consumed by Saharawi diabetic women.

	Healthy Dietary Pattern	Unhealthy Dietary Pattern
Grain cereals	−0.273	0.110
Bread, pasta, rice, couscous	0.095	**0.807**
Potatoes	−0.040	0.006
Fried potatoes	−0.043	−0.076
Onions, tomatoes, and carrots	0.027	**0.798**
Other vegetables	**0.636**	0.101
Olive oil	**0.585**	−0.220
Sunflower oil	−0.191	**0.671**
Animal fats	**−0.365**	0.290
Fresh fruit	**0.584**	−0.156
Dried fruit	0.205	**0.466**
Red meat	**−0.568**	**0.466**
White meat	**0.705**	0.247
Eggs	**0.480**	−0.049
Low-fat milk	**0.339**	−0.190
Camel’s, cow’s, and goat’s milk and dairy products	**0.518**	0.099
Legumes	**0.389**	0.232
Canned fish	0.264	0.108
Fresh fish	−0.088	**−0.305**
Sugar, sweets, and beverages	−0.132	**0.480**
Explained variance (%)	15.3	14.3

Factor loadings characterizing each dietary pattern. Factor loadings with an absolute value ≥ 0.3 are reported in bold font.

**Table 4:** Association of healthy and unhealthy dietary patterns with the nutritional status and the glycemic profile.

	Healthy Dietary Pattern			Unhealthy Dietary Pattern		
	T1	T2	T3	T1	T2	T3
Nutritional status *						
BMI	Reference	0.76	1.62	Reference	0.66	−0.65
		[−1.95, 3.47]	[−1.18, 4.43]		[−2.09, 3.40]	[−3.45, 2.15]
WC	Reference	−0.69	1.00	Reference	4.01	−0.47
		[−6.66, 5.29]	[−5.17, 7.16]		[−1.94, 9.96]	[−6.50, 5.56]
WHR	Reference	0.00	0.01	Reference	0.03	0.01
		[−0.06, 0.05]	[−0.05, 0.07]		[−0.02, 0.09]	[−0.04, 0.07]
BF%	Reference	0.21	−0.28	Reference	1.35	1.90
		[−1.76, 2.17]	[−2.35, 1.79]		[−0.61, 3.31]	[−0.12, 3.91]
Glycemic profile ^†^						
HbA1c (%)	Reference	0.39	−0.06	Reference	−0.36	0.50
		[−0.65, 1.42]	[−1.12, 0.99]		[−1.38, 0.66]	[−0.55, 1.55]
HOMA	Reference	−0.05	−0.49	Reference	−0.03	2.49 *
		[−2.22, 2.11]	[−2.74, 1.76]		[−2.06, 1.99]	[0.41, 4.57]
Insulin (mU/mL)	Reference	0.00	−0.90	Reference	0.89	4.52 *
		[−4.18, 4.18]	[−5.24, 3.44]		[−3.08, 4.87]	[0.44, 8.60]
Glucose (mg/dL)	Reference	1.71	−6.93	Reference	−17.86	6.58
		[−37.30, 40.73]	[−46.87, 33.02]		[−56.56, 20.84]	[−33.21, 46.36]

Values are linear regression coefficient and 95% CI in brackets. * Model adjusted for age and years passed from the diagnosis of diabetes. ^†^ Model adjusted for age, years passed from the diagnosis of diabetes, and BMI.
